# Tamoxifen treatment fails to improve muscle dysfunction in a model of recessive *RYR1*-linked centronuclear myopathy

**DOI:** 10.1242/dmm.052462

**Published:** 2025-12-29

**Authors:** Charlotte Gineste, David Reiss, Jocelyn Laporte

**Affiliations:** Department of Translational Medicine and Neurogenetics, Institut de Génétique et de Biologie Moléculaire et Cellulaire (IGBMC), Inserm U1258, Cnrs UMR7104, Strasbourg University, Illkirch 67404, France

**Keywords:** Congenital myopathy, Pharmacotherapy, Force production, Muscle atrophy, Mouse model, Internalized nuclei

## Abstract

Centronuclear myopathies (CNMs) are rare congenital muscle disorders with no effective treatment. Previous studies showed that tamoxifen improved muscle function in mice modeling CNMs caused by variants in *MTM1*, *BIN1* and *DNM2*. Here, we investigated whether tamoxifen administration improves muscle function and pathology in the severe recessive *Ryr1*^TM/indel^ mouse model of *RYR1*-related CNM. Contractile performance, histological analyses and protein levels were assessed in *Ryr1*^TM/indel^ mice and control littermates (wild type) treated with either a tamoxifen-enriched diet (65 mg/kg of food) or a control diet for 5 weeks, beginning at 3 weeks of age. *Ryr1*^TM/indel^ mice displayed muscle weakness, reduced myofiber size and a high number of fibers with nuclei in abnormal position, regardless of the treatment. Force production during repeated contractions was reduced in tamoxifen-treated *Ryr1*^TM/indel^ mice compared to that in untreated *Ryr1*^TM/indel^ mice. The levels of CNM proteins (DNM2 and BIN1) were unchanged following the treatment. Tamoxifen did not improve muscle dysfunction, atrophy or histological hallmarks in *Ryr1*^TM/indel^ mice. Our data indicate that tamoxifen supplementation is not beneficial and may negatively impact muscle function in this model of CNM, suggesting limited therapeutic value for patients with *RYR1* mutations.

## Teaser

**Summary:** No improvement in muscle phenotype is observed in recessive *Ryr1*-linked centronuclear myopathy mice following tamoxifen administration.

## INTRODUCTION

Centronuclear myopathies (CNMs) are neuromuscular disorders resulting in several clinical manifestations including muscle weakness, muscle hypotonia and muscle atrophy. The main histological hallmark of the pathology is the mispositioning of organelles and the abnormal centralization of nuclei in muscle fibers without obvious degenerative process (Jungbluth et al., 2008; Gomez-Oca et al., 2021). Congenital myopathies with mislocalized nuclei include CNMs and are genetically heterogeneous diseases caused by mutations in at least seven genes (*MTM1*, *DNM2*, *BIN1*, *RYR1*, *TTN*, *SPEG* and *ZAK*), with *MTM1*, *DNM2*, *BIN1* and *RYR1* being the most frequently associated (Jungbluth et al., 2008). The main forms of CNM are divided according to the mode of inheritance and the mutation. The X-linked recessive form of CNM, also called myotubular myopathy (i.e. XLMTM), is associated with mutations in *MTM1* and is the most severe form of the disease (Laporte et al., 1996). The main autosomal dominant form is related to mutations in *DNM2*, with a clinical severity ranging from a mild adult form to a severe neonatal form depending on the mutation (Bitoun et al., 2007; Bohm et al., 2012). The most frequent autosomal recessive form is caused by mutations in *RYR1*, and is typically associated with a severe clinical phenotype and early onset of the disease (Amburgey et al., 2013; Jungbluth et al., 2007; Wilmshurst et al., 2010; Bevilacqua et al., 2011). Mutations in *BIN1* are either dominantly or recessively inherited. The recessive form of *BIN1* is associated with early childhood onset, whereas the dominant form is associated with a progressive adulthood onset myopathy (Bohm et al., 2014; Nicot et al., 2007). Myotubularin (MTM1), encoded by the *MTM1* gene, is a phosphoinositide phosphatase regulating membrane trafficking (Gomez-Oca et al., 2021). *DNM2* encodes dynamin 2 (DNM2), a mechanoenzyme involved in membrane fission through its GTPase activity (Antonny et al., 2016; Ferguson and De Camilli, 2012). Amphiphysin 2 (BIN1), encoded by *BIN1*, is implicated in membrane curvature and remodeling (Lee et al., 2002; Peter et al., 2004). *RYR1* encodes the Ca^2+^ release channel ryanodine receptor 1 protein (RYR1) located at the sarcoplasmic reticulum (SR) membrane of the cisternae at the triad (Jungbluth et al., 2018; Santulli et al., 2017). In skeletal muscle, these proteins play a structurally or functionally fundamental role at the triad through the formation and maintenance of T-tubules, and Ca^2+^ release from the SR to the cytosol.

Currently, there is no approved therapy for any form of CNM. Several strategies have been explored to cure CNMs by using gene therapy to correct the underlying genetic anomalies, or by using pharmacological compounds to treat or prevent muscle defects (Gomez-Oca et al., 2021). Gene-based therapies mainly reduce DNM2 protein levels or increase MTM1 or BIN1 protein levels (Tasfaout et al., 2017; Lionello et al., 2019; Giraud et al., 2023; Buj-Bello et al., 2008). Among the tested drugs, tamoxifen and vitamin K3 demonstrated therapeutic efficiency in murine models of CNM (Gineste et al., 2023; Swamynathan et al., 2024; Gayi et al., 2018; Maani et al., 2018). Importantly, most of these genetic and pharmacological treatments were validated in preclinical models of several forms of CNM and thus represent common therapeutic strategies. Particularly, tamoxifen showed positive effects in mouse models for three genetic forms of the disease (*MTM1*-, *DNM2*- and *BIN1*-related CNM). Although the mechanism of action of tamoxifen in muscle is not known, it was hypothesized that its effect was mediated through estrogen receptor alpha (ERα; encoded by *ESR1*). Tamoxifen probably acts on skeletal muscle through non-genomic effects considering the membranous expression of ERα in muscle fibers and the absence of significant change in the transcriptome of the muscle from tamoxifen-treated CNM animals (Gineste et al., 2023; Maani et al., 2018). In addition, it was demonstrated that tamoxifen decreased the abnormally high level of DNM2, which is involved in the disease process, suggesting that the positive effect of tamoxifen in CNM mice was, at least in part, related to reduction in DNM2 (Gineste et al., 2023; Maani et al., 2018).

Unfortunately, therapeutic strategies have never been assessed for the *RYR1* form of CNM until recently owing to the lack of a murine model of recessive *RYR1*-related CNM. Brennan et al. (2019) generated the first mouse model exhibiting a severe and early-onset recessive *RYR1*-related myopathy. This genetically engineered mouse model is compound heterozygous for a substitution (T4709M missense mutation, commonly reported in patients with recessive myopathy linked to *RYR1*) and a deletion (16 bp frameshift deletion) and named the *Ryr1*^TM/indel^ mouse model. *Ryr1*^TM/indel^ mice display progressive muscle weakness, reduced myofiber size and central nuclei within muscle fibers, i.e. the main signs of CNM. Thus, the *Ryr1*^TM/indel^ mouse represents a suitable model for preclinical testing of potential therapies for CNM related to *RYR1*. Considering that the *RYR1*-related form is one of the main genetic forms of the disease, it is of utmost importance to investigate therapeutic strategies for *RYR1*-related CNM. We previously demonstrated that tamoxifen administration resulted in phenotypic improvements in mouse models of *MTM1*-, *DNM2*- and *BIN1*-related CNM (Gineste et al., 2023; Gayi et al., 2018; Maani et al., 2018). Thus, we hypothesized that *RYR1*-related CNM can be effectively treated with tamoxifen. The choice of tamoxifen is of high interest for fast clinical translation considering that it is an already approved drug that may be repurposed for the *RYR1*-related form of CNM. The purpose of this study was to preclinically investigate whether tamoxifen administration improves the phenotype of the *Ryr1*^TM/indel^ mouse model of CNM linked to *RYR1*.

## RESULTS

### Muscle exposure to tamoxifen does not prevent the loss of body mass and muscle mass in *Ryr1*^TM/indel^ mice

**Fig. 1.**
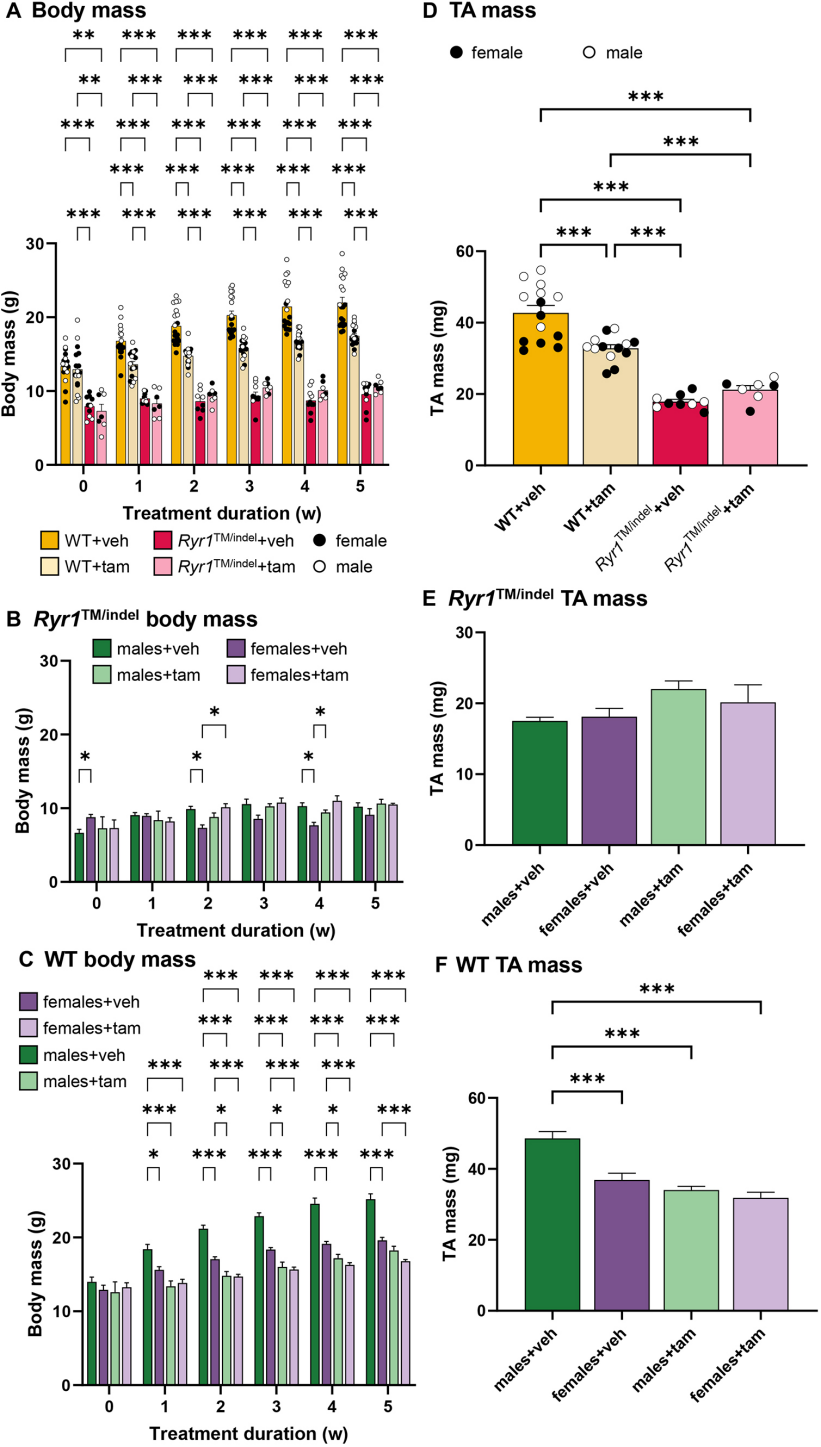
**Tamoxifen did not increase body mass or muscle mass in *Ryr1*^TM/indel^ mice.** (A) Body mass before and during treatment intervention that was started at 3 weeks of age in *Ryr1*^TM/indel^ and wild-type (WT) mice. tam, tamoxifen; veh, vehicle. (B,C) Body mass of male and female *Ryr1*^TM/indel^ (B) and WT (C) mice before and during treatment intervention that was started at 3 weeks of age. w, weeks. (D) Mass of the tibialis anterior (TA) in the *Ryr1*^TM/indel^ and WT groups. (D) TA mass in *Ryr1*^TM/indel^ and WT mice before and during treatment intervention that was started at 3 weeks of age. (E,F) TA mass of male and female *Ryr1*^TM/indel^ (E) and WT (F) mice before and during treatment intervention that was started at 3 weeks of age. (D) Mass of TA in *Ryr1*^TM/indel^ and WT groups. Data presented as individual values and mean±s.e.m. in A and D, and as mean±s.e.m. in B, C, E and F. *P*-values obtained with mixed-effects analysis or two-way ANOVA with repeated measures on time with Tukey's post hoc test (A-C) or with one-way ANOVA with Tukey's post hoc test (B,E,G). *P<0.05, **P<0.01 and ***P<0.001.

To assess a potential beneficial effect of tamoxifen supplementation in the mouse model of *RYR1*-related CNM, we compared untreated and tamoxifen-treated *Ryr1*^TM/indel^ mice with untreated and tamoxifen-treated wild-type (WT) mice. Mice were supplemented with tamoxifen for 5 weeks from 3 weeks of age to 8 weeks of age. Both *Ryr1*^TM/indel^ groups had significantly lower body mass than that of untreated WT mice from the start to the end of the treatment period (−47% at week 0 and −53% at week 5 post treatment; *P*<0.05) (Fig. 1A). Untreated and tamoxifen-treated *Ryr1*^TM/indel^ mice had similar body mass throughout the treatment intervention [6.8±0.4 g (mean±s.e.m.) for untreated *Ryr1*^TM/indel^ versus 7.3±0.8 g for tamoxifen-treated *Ryr1*^TM/indel^ at week 0 (before treatment start at 3 weeks of age), and 9.9±0.5 g for untreated *Ryr1*^TM/indel^ versus 10.6±0.3 g for tamoxifen-treated *Ryr1*^TM/indel^ at week 5 post treatment; *P*>0.05]. Untreated *Ryr1*^TM/indel^ females had higher body mass than untreated *Ryr1*^TM/indel^ males before tamoxifen administration (+30%) (Fig. 1B). After 1 week of treatment, both groups showed similar body mass, whereas from 2 weeks of tamoxifen administration body mass of *Ryr1*^TM/indel^ males was higher than that of *Ryr1*^TM/indel^ females (+30%). Body mass of tamoxifen-treated *Ryr1*^TM/indel^ males and females was similar throughout the treatment period. Of note, body mass was significantly decreased in tamoxifen-treated WT mice compared to that in untreated WT mice from 1 week of treatment and remained lower until the last week of treatment (13.6±0.4 g versus 17.5±0.5 g, respectively, at week 1 post treatment, and 17.5±0.3 g versus 21.9±0.7 g, respectively, at week 5 post treatment; *P*<0.05). WT males and females treated with tamoxifen showed significantly reduced body mass compared to that of untreated sex-matched mice from the start of treatment administration (Fig. 1C). Body mass reduction was higher in males (−27% at 1 and 5 weeks post treatment) than in females (−11% at 1 week post treatment and −15% at 5 weeks post treatment). Tamoxifen-treated WT males and females had similar body mass throughout the treatment period.

To confirm tamoxifen intake in mice and that the supplemented tamoxifen reached the muscles, we calculated the tamoxifen intake every week, and we measured the level of its metabolites in gastrocnemius (Gas) at 8 weeks of age. The amount of tamoxifen intake was estimated based on the amount of food intake. Food intake per mouse or per kg of body mass was similar between *Ryr1*^TM/indel^ groups, WT groups and tamoxifen-treated groups (Fig. S1A,B). Consequently, tamoxifen intake per mouse and per kg of body mass was similar in WT and *Ryr1*^TM/indel^ mice (Fig. S1C,D). Accordingly, the levels of tamoxifen metabolites in muscle from tamoxifen-treated WT and *Ryr1*^TM/indel^ mice were not significantly different (Table S1).

Tibialis anterior (TA), soleus (Sol) and Gas muscle masses were significantly reduced in both *Ryr1*^TM/indel^ groups compared to those in WT mice (−50%, −55% and −55%, respectively; *P*<0.05). Masses of TA, Sol and Gas were unchanged in *Ryr1*^TM/indel^ mice following tamoxifen administration (Fig. 1D; Fig. S2). No significant difference was observed between *Ryr1*^TM/indel^ males and females, regardless of the treatment (Fig. 1E). Both TA mass and Sol mass were significantly lower in tamoxifen-treated WT mice than those in untreated WT mice (−25%; *P*<0.05). Mass of the TA was lower in tamoxifen-treated WT males than that in untreated WT males (−30%), whereas no difference was observed for WT females (Fig. 1F). Overall, tamoxifen supplementation did not improve the reduced body and muscle masses of *Ryr1*^TM/indel^ mice.

### Altered spontaneous locomotor activity of *Ryr1*^TM/indel^ mice persists despite tamoxifen administration

**Fig. 2.**
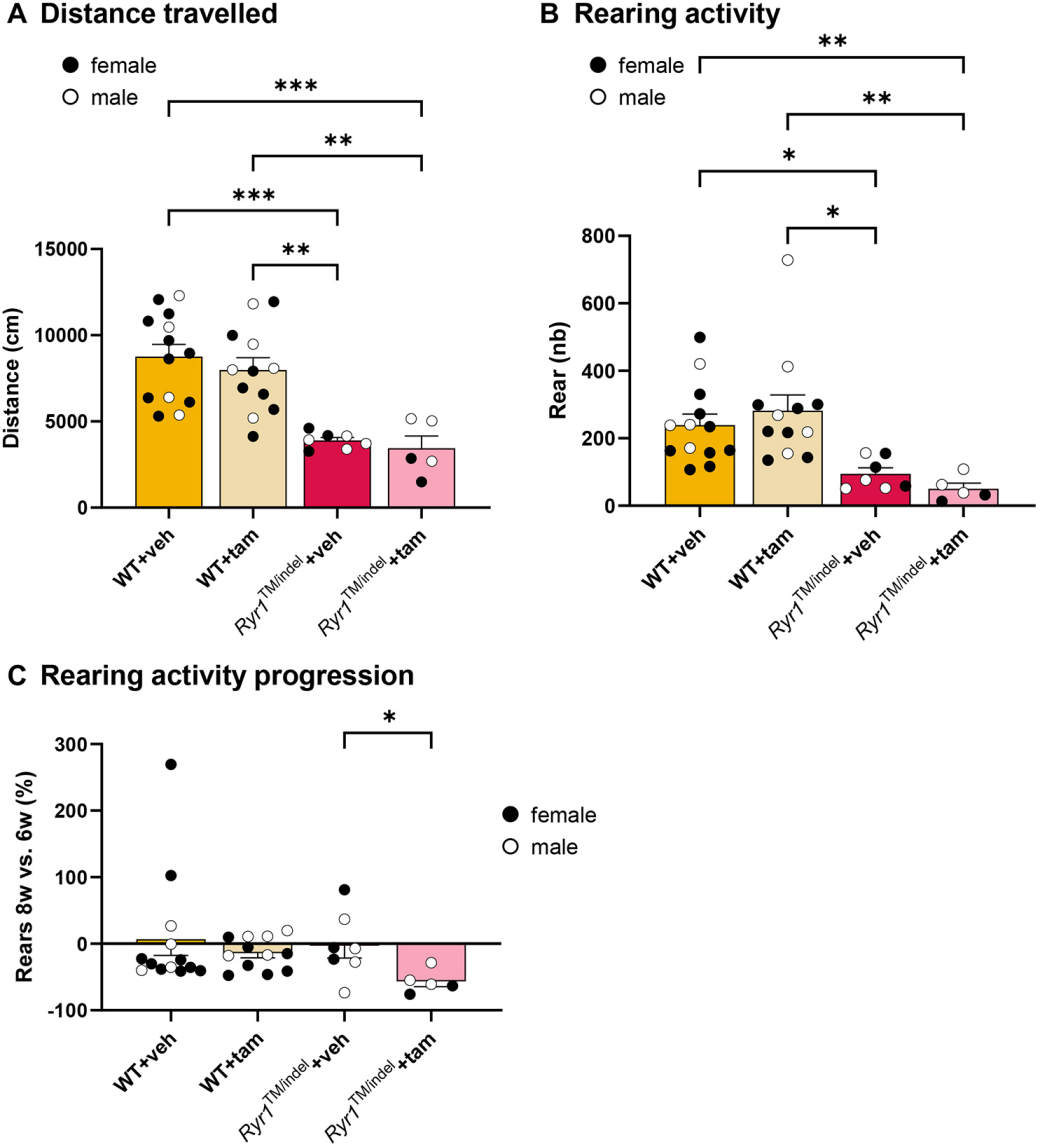
**Tamoxifen did not improve spontaneous locomotor activity in *Ryr1*^TM/indel^ mice.** (A,B) Distance covered (A) and number of rears (B) during a 30-min period in *Ryr1*^TM/indel^ and WT groups at 8 weeks. nb, number. (C) Changes in rearing activity 3 and 5 weeks after the start of treatment intervention, i.e. at 6 weeks and 8 weeks of age, in *Ryr1*^TM/indel^ and WT groups. Data presented as individual values and mean±s.e.m. *P*-values obtained with one-way ANOVA with Tukey's post hoc test (A) or Kruskal–Wallis with Dunn's post hoc test (B,C). *P<0.05, **P<0.01 and ***P<0.001.

In addition to muscle mass reduction, mutations in *Ryr1* led to decreased motor performance. Both untreated and treated *Ryr1*^TM/indel^ groups displayed decreased distance traveled at 6 weeks and 8 weeks compared to WT groups (5440±474 cm versus 9834±404 cm, respectively, at 6 weeks, and 3706±303 cm versus 8382±501 cm, respectively, at 8 weeks) (Fig. S3A and Fig. 2A, respectively). Rearing activity was also significantly lower at 6 and 8 weeks in *Ryr1*^TM/indel^ groups than in WT groups [114±14 number (nb) versus 293±25 nb at 6 weeks and 76±14 nb versus 260±28 nb at 8 weeks] (Fig. 2B; Fig. S3B). Tamoxifen-treated *Ryr1*^TM/indel^ mice showed a reduced number of rears at 8 weeks compared to that at 6 weeks (−57±8%), whereas the number of rears was similar in other groups between 6 and 8 weeks (Fig. 2C). Therefore, rearing activity was significantly lowered over time in tamoxifen-treated *Ryr1*^TM/indel^ mice compared to that in untreated *Ryr1*^TM/indel^ mice. However, no significant difference was observed over time between the four groups for the amount of distance traveled (Fig. S3C). Taken together, we conclude that the *Ryr1*^TM/indel^ mice display a strong decrease in motor performance, without a positive effect of tamoxifen supplementation.

### Reduced muscle force but unaltered fatigue resistance in *Ryr1*^TM/indel^ mice, independent of tamoxifen treatment

Because *Ryr1* mutations are associated with impaired motor performance, we next investigated whether this impairment results from altered muscle force production. To do this, we measured muscle force output and fatigue resistance in the TA muscle using *in situ* force transducer recordings.

**Fig. 3.**
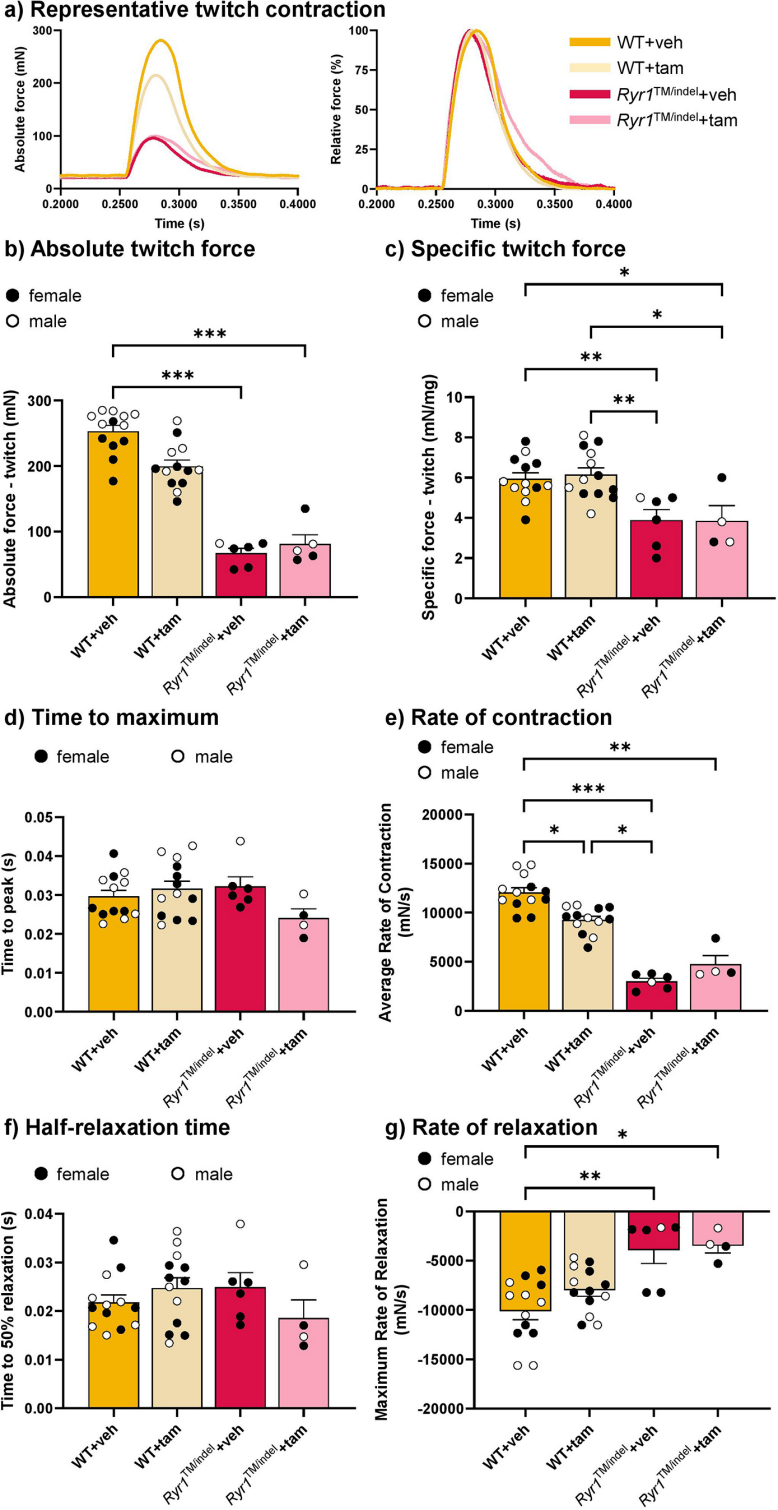
**Twitch muscle force was reduced in *Ryr1*^TM/indel^ mice regardless of the treatment.** (A) Representative traces of absolute twitch force (left) and relative twitch force (right). (B,C) *In situ* absolute (B) and specific (C) twitch force of the TA following 5 weeks of exposure to a standard diet or a tamoxifen-enriched diet in *Ryr1*^TM/indel^ and WT mice. (D-G) Contractile properties of the twitch contraction [time to maximum (D), rate of contraction (E), half relaxation time (F) and rate of relaxation (G)] in *Ryr1*^TM/indel^ and WT mice at the end of the 5 weeks treatment protocol. Data presented as individual values and mean±s.e.m. *P*-values obtained with one-way ANOVA with Tukey's post hoc test (C,D,F) or Kruskal–Wallis with Dunn's post hoc test (B,E,G). *P<0.05, **P<0.01 and ***P<0.001.

The raw and specific twitch forces were significantly reduced in *Ryr1*^TM/indel^ mice compared to those in WT mice (−75% and −35%, respectively) (Fig. 3A-C). Time to maximum was similar between groups (Fig. 3D), whereas the rate of contraction was reduced in all groups compared to that in untreated WT mice (Fig. 3E). No difference was observed in the rate of contraction between *Ryr1*^TM/indel^ groups. The half relaxation time post contraction was not different between groups (Fig. 3F). The rate of relaxation was decreased in both *Ryr1*^TM/indel^ groups compared to that in the untreated WT group and similar between *Ryr1*^TM/indel^ groups (Fig. 3G).

**Fig. 4.**
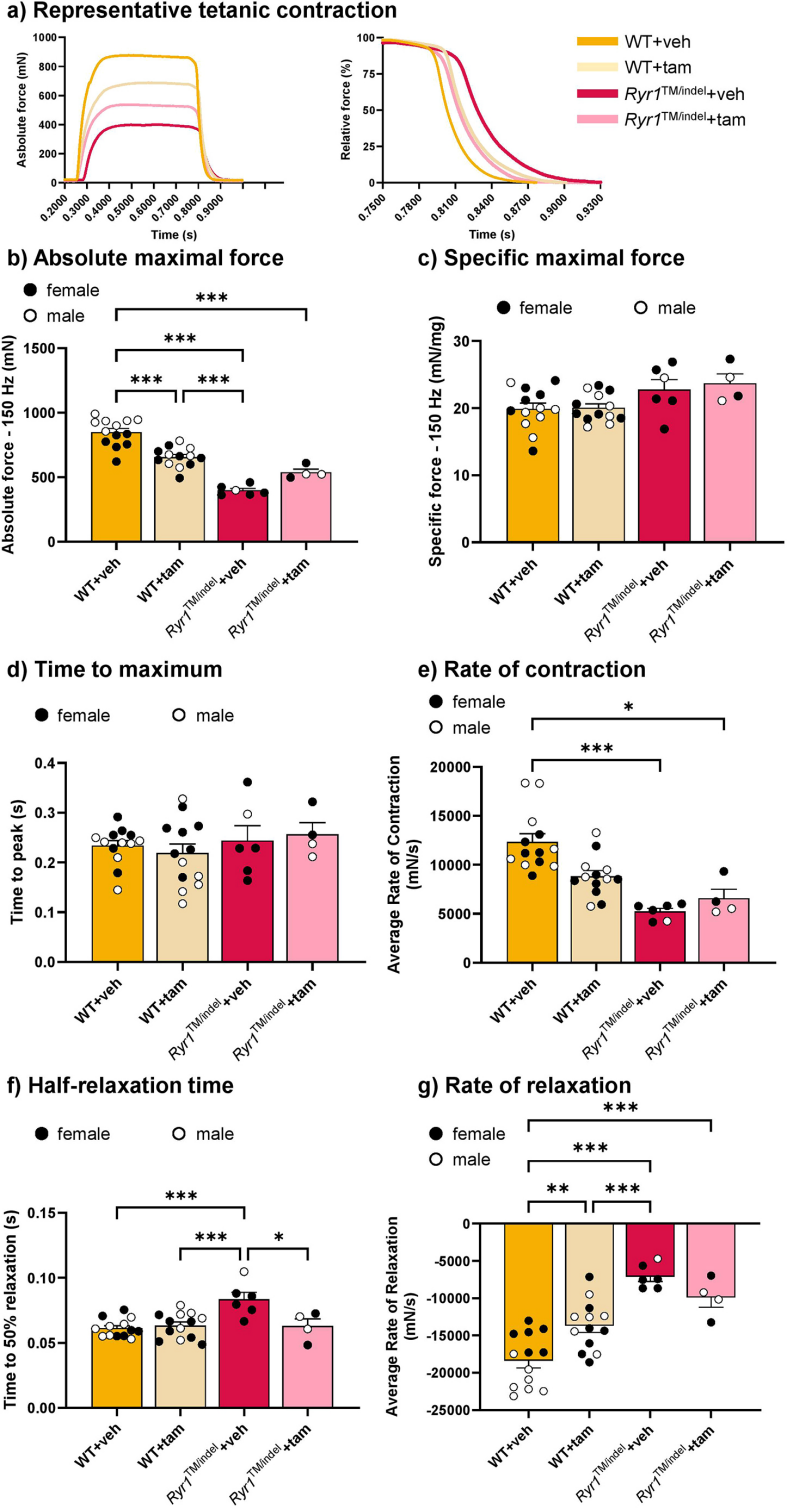
**Muscle weakness in *Ryr1*^TM/indel^ mice persists despite tamoxifen administration.** (A) Representative traces of absolute maximal force (left) and relaxation post maximal force (right). (B,C) *In situ* absolute (B) and specific (C) maximal tetanic force of the TA following 5 weeks of exposure to a standard diet or a tamoxifen-enriched diet in *Ryr1*^TM/indel^ and WT mice. (D-G) Contractile properties of the maximal tetanic contraction obtained at 150 Hz [time to maximum (D), rate of contraction (E), half relaxation time (F) and rate of relaxation (G)] in *Ryr1*^TM/indel^ and WT mice at the end of the 5 weeks treatment protocol. Data presented as individual values and mean±s.e.m. *P*-values obtained with one-way ANOVA with Tukey's post hoc test (A-D,F,G) or Kruskal–Wallis with Dunn's post hoc test (E). *P<0.05, **P<0.01 and ***P<0.001.

The raw maximal tetanic force (150 Hz) was significantly lower in *Ryr1*^TM/indel^ mice than in WT mice, regardless of the treatment (−40%) (Fig. 4A,B). Tamoxifen-treated WT mice showed a significant decrease in raw maximal tetanic force in comparison with that in the untreated WT group (−23%), supporting a detrimental effect of tamoxifen on this mouse group. Conversely, specific maximal tetanic force was not different between groups, showing that the decrease in maximal force production was mostly linked to the decrease in muscle mass (*P*>0.05) (Fig. 4C). Time to reach maximum was identical in WT and *Ryr1*^TM/indel^ groups, regardless of the treatment (Fig. 4D). A reduced rate of contraction was observed in both *Ryr1*^TM/indel^ groups compared to that in the untreated WT group (Fig. 4E). A significant increase in the half relaxation time was observed in the untreated *Ryr1*^TM/indel^ group compared to that in the WT groups, and a significant decrease was reported in tamoxifen-treated *Ryr1*^TM/indel^ mice compared to that in the untreated *Ryr1*^TM/indel^ groups (Fig. 4F). The rate of relaxation was significantly lower in all groups compared to that in the untreated WT group and similar between *Ryr1*^TM/indel^ mice (Fig. 4G).

**Fig. 5.**
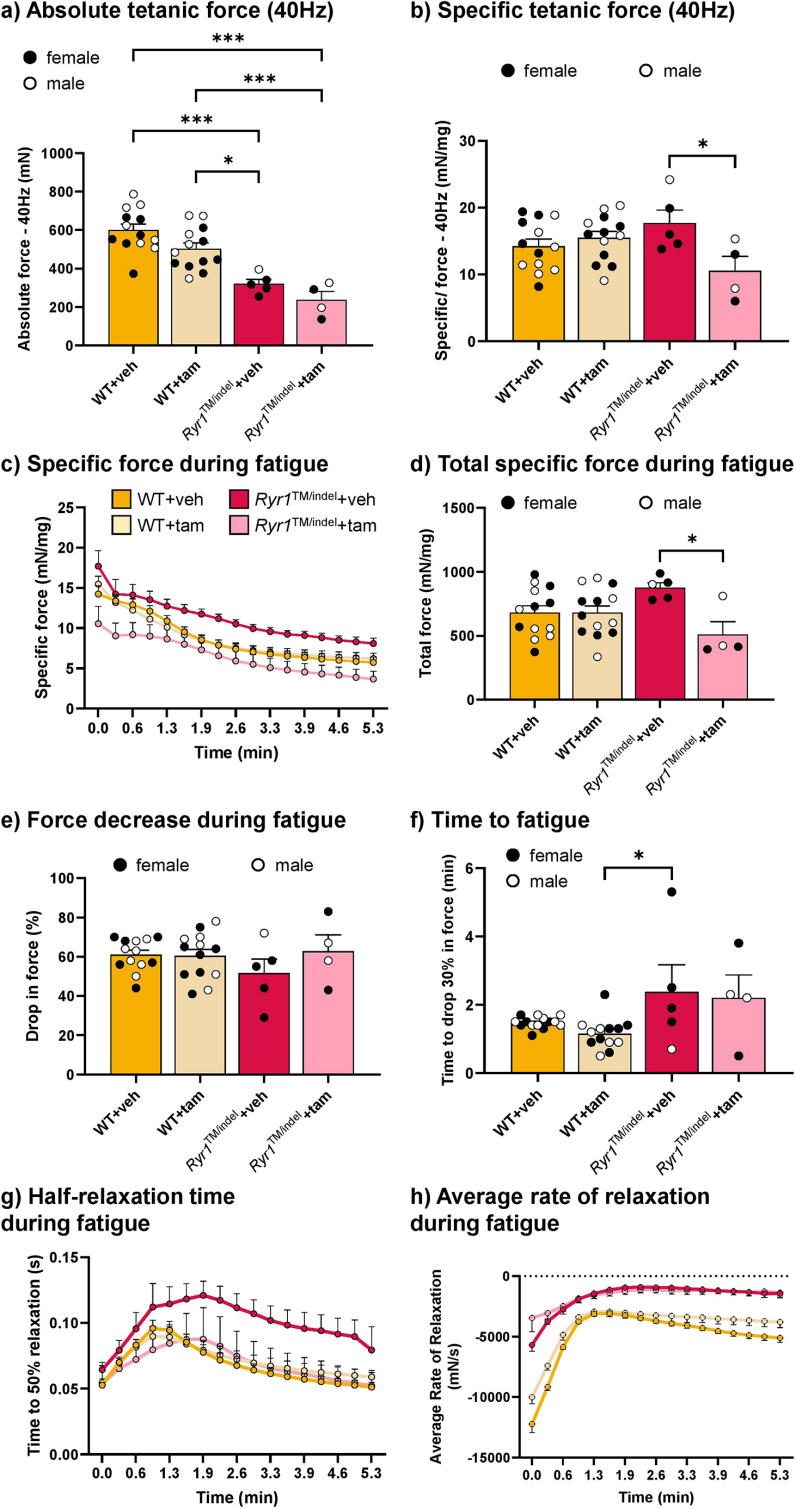
**Fatigue resistance was not altered by the *Ryr1*^TM/indel^ mutation or by tamoxifen administration.** (A,B) *In situ* absolute (A) and specific (B) tetanic force of the TA obtained at 40 Hz following 5 weeks of exposure to a standard diet or a tamoxifen-enriched diet in *Ryr1*^TM/indel^ and WT mice. (C,D) *In situ* specific force (C) and total force (D) of the TA during fatigue in *Ryr1*^TM/indel^ and WT mice at the end of the 5 weeks treatment protocol, i.e. at 8 weeks of age. (E,F) Quantification of the drop in force (E) and time to fatigue (F) in *Ryr1*^TM/indel^ and WT mice during fatigue at the end of the treatment protocol. (G,H) Half relaxation time (G) and rate of relaxation (H) during fatigue in *Ryr1*^TM/indel^ and WT mice during fatigue at the end of the treatment protocol. Data presented as individual values and mean±s.e.m. in A, B and D-F, and as mean±s.e.m. in C, G and H. *P*-values obtained with one-way ANOVA with Tukey's post hoc test (A,B,E,F), Kruskal–Wallis with Dunn's post hoc test (D), or two-way ANOVA with repeated measures with Tukey's post hoc test (C,G,H). *P<0.05 and ***P<0.001.

Fatiguing protocol consisted in repetitive submaximal contractions (40 Hz). Raw force produced during the first contraction of the fatigue protocol was significantly decreased in the *Ryr1*^TM/indel^ groups compared to that in the WT groups (Fig. 5A), whereas specific force was not different from that in the WT groups (Fig. 5B). However, specific force was significantly lower in tamoxifen-treated *Ryr1*^TM/indel^ mice than that in untreated *Ryr1*^TM/indel^ mice.

Force production during fatigue was lower from the start to the end of exercising in *Ryr1*^TM/indel^ mice following tamoxifen administration compared to that in untreated *Ryr1*^TM/indel^ mice, whereas no difference from that in WT mice was observed (Fig. 5C). Accordingly, the total force produced during the repetitive contractions was significantly lower in the tamoxifen-treated *Ryr1*^TM/indel^ group than in the in untreated *Ryr1*^TM/indel^ group (−45%). Total force produced during the repetitive stimulations was similar in WT groups (Fig. 5D). Nevertheless, the drop in force during fatigue was not different between groups (∼60%) (Fig. 5E). The time to fatigue was not different in all groups compared to that in the untreated WT group (Fig. 5F). During fatigue, half relaxation time was similar in untreated WT, tamoxifen-treated WT and tamoxifen-treated *Ryr1*^TM/indel^ mice over time. However, half relaxation time was higher throughout the fatigue protocol in untreated *Ryr1*^TM/indel^ mice than in the other groups (Fig. 5G). The rate of relaxation during fatigue was higher in WT groups than in *Ryr1*^TM/indel^ groups at the start of the fatigue protocol. Rate decreased over time in the WT groups (Fig. 5H). In conclusion, *Ryr1*^TM/indel^ mice displayed a decrease in muscle force related to reduced muscle mass. Tamoxifen supplementation did not improve the muscle production of these mice and even decreased the overall force produced by their TA muscles during repetitive contractions.

### Histological abnormalities observed in untreated and tamoxifen-treated *Ryr1*^TM/indel^ mice

**Fig. 6.**
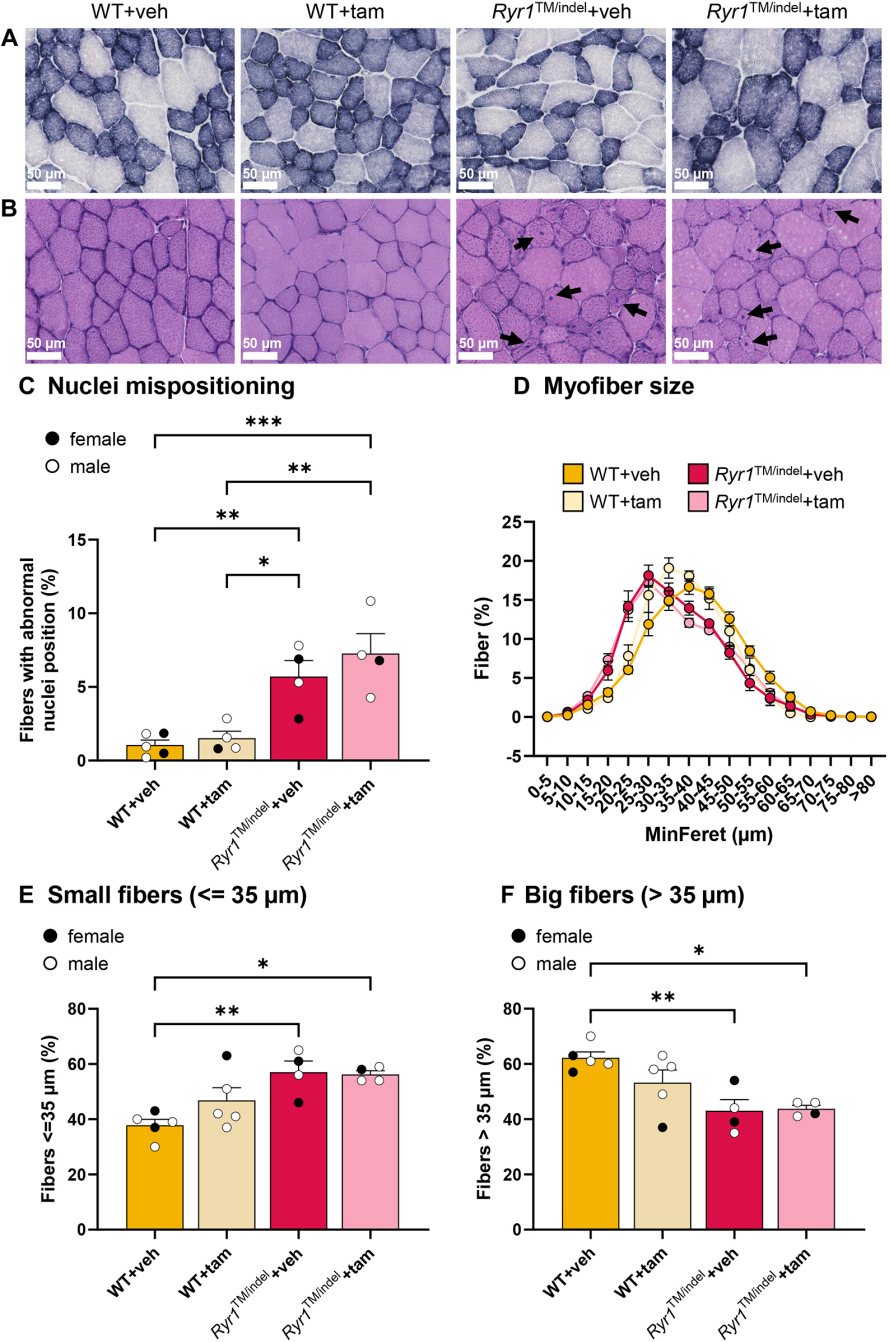
**Tamoxifen did not reverse nuclei internalization or atrophy of muscle fibers.** (A,B) Representative images of succinate dehydrogenase activity (A) and Hematoxylin-Eosin staining (B) of TA muscle sections from untreated and tamoxifen-treated *Ryr1*^TM/indel^ and WT mice. Arrows show abnormally positioned nuclei. (C) Percentage of fibers with mislocalized nuclei in TA muscle of *Ryr1*^TM/indel^ and WT mice. (D) Minimum feret (MinFeret) perimeter distribution of TA fibers in *Ryr1*^TM/indel^ and WT mice that were tamoxifen treated or untreated for 5 weeks. (E,F) Quantification of muscle fiber size with MinFeret diameter ≤35 μm (E) and >35 μm (F). Data presented as individual values and mean±s.e.m. in C, E and F, and as mean±s.e.m. in D. *P*-values obtained with one-way ANOVA with Tukey's post hoc test (C,E,F). *P<0.05, **P<0.01 and ***P<0.001.

In addition to muscle weakness, *RYR1*-related CNM is defined by histological hallmarks including decreased myofiber size and organelle mispositioning. *Ryr1*^TM/indel^ mice, regardless of the treatment, did not display any mitochondrial anomalies as monitored with succinate dehydrogenase (SDH) staining (Fig. 6A). However, a significant increase in internalized nuclei was observed in 5-7% of muscle fibers in both *Ryr1*^TM/indel^ groups compared to those in WT (Fig. 6B,C). WT mice did not display any increase in nuclei mislocalization with tamoxifen treatment.

Myofiber size was similarly reduced in *Ryr1*^TM/indel^ groups compared to that in untreated WT mice (Fig. 6D). Accordingly, the number of small fibers with a diameter ≤35 µm was significantly higher in *Ryr1*^TM/indel^ groups than in untreated WT mice (57±2% versus 42±3%, respectively; *P*<0.05), whereas the amount of large fibers with a diameter >35 µm was significantly lower (43±2% versus 58±3%, respectively; *P*<0.05) (Fig. 6E,F). Myofiber size was comparable in WT groups and *Ryr1*^TM/indel^ groups. Overall, the *Ryr1*^TM/indel^ mice reproduced the main histological signs of *RYR1*-related CNM, reduction in myofiber size and mislocalized nuclei, which were not rescued by tamoxifen treatment.

### The levels of proteins involved in CNMs are not modified by tamoxifen treatment

**Fig. 7.**
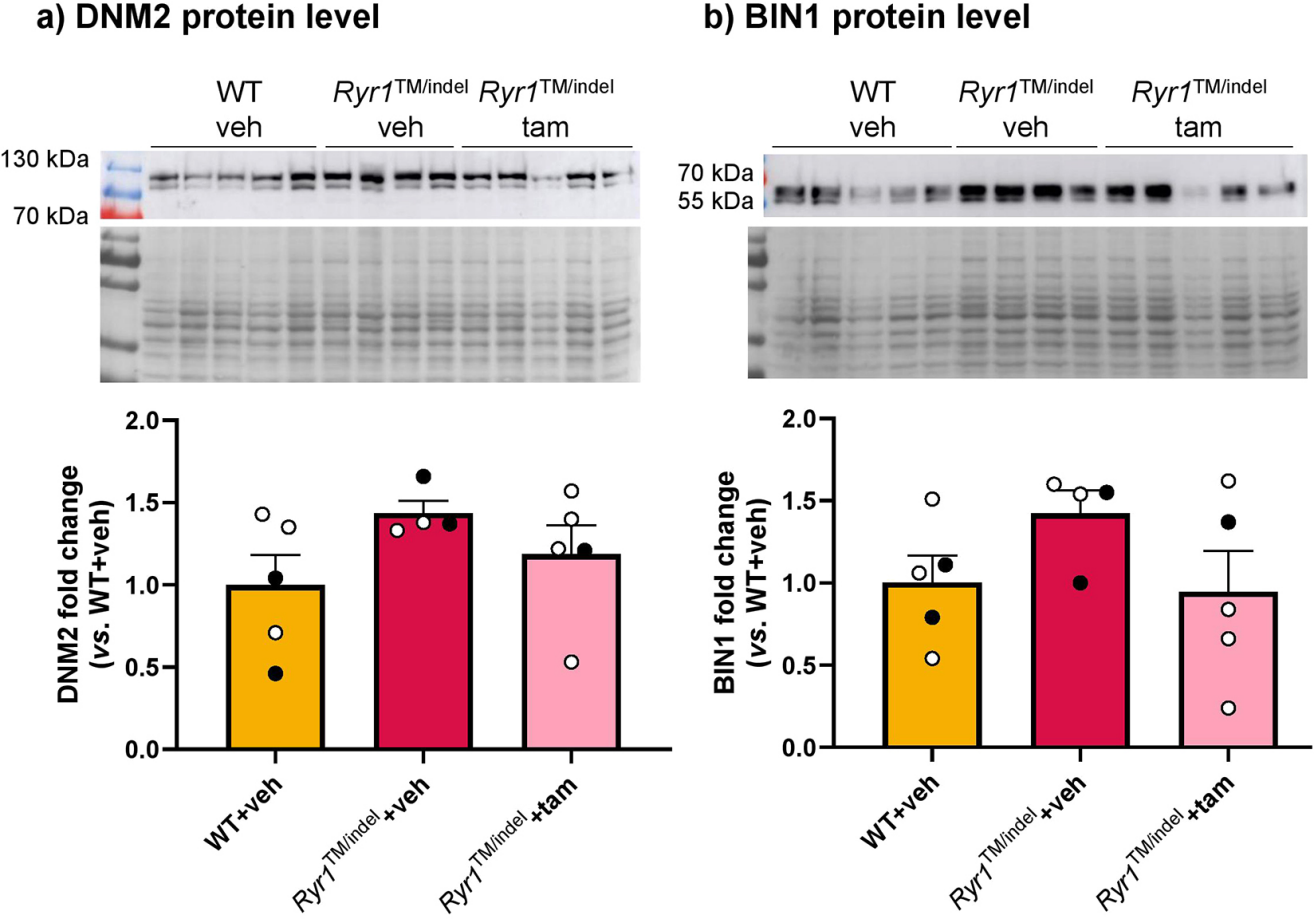
**The levels of proteins involved in the centronuclear myopathy disease process were not affected by tamoxifen treatment.** (A,B) Representative western blot and quantification of DNM2 (A) and BIN1 (B) in TA muscles of 8-week-old untreated WT mice and *Ryr1*^TM/indel^ mice that were untreated and treated with tamoxifen. Data presented as individual values and mean±s.e.m. *P*-values obtained with one-way ANOVA with Kruskal–Wallis with Dunn's post hoc test.

As DNM2 and BIN1 proteins were found to be increased in other genetic forms of CNM linked, for example, to *MTM1* mutations and subsequently normalized with tamoxifen treatment (Gayi et al., 2018; Maani et al., 2018), we assessed their levels in the muscles of *Ryr1*^TM/indel^ mice. DNM2 and BIN1 levels did not significantly increase in untreated *Ryr1*^TM/indel^ mice compared to those in untreated WT mice (Fig. 7). Tamoxifen supplementation did not significantly impact the levels of these proteins in *Ryr1*^TM/indel^ mice.

## DISCUSSION

In the present study, we characterized the *Ryr1*^TM/indel^ mice modeling *RYR1*-related CNM and assessed the effects of tamoxifen administration on muscle structure and function. Our results showed no improvement of the phenotype at functional, histological and molecular levels, which contrast with previous data on other preclinical models of CNM linked to mutations in other genes.

### Characterization and physiological findings in the *Ryr1*^TM/indel^ mouse model of *RYR1*-related CNM

We first characterized the *Ryr1*^TM/indel^ mice modeling *RYR1*-related CNM. We observed a strong phenotype *in vivo* at 6 weeks and 8 weeks characterized by a ∼40-60% reduction in distance traveled and ∼60-70% reduction in rearing activity compared to those in WT mice. At 8 weeks, both the absolute twitch and tetanic forces of the TA were significantly lower in *Ryr1*^TM/indel^ mice than those in WT mice. These decreases in forces were likely to be related to the lowered amplitude of Ca^2+^ release reported in muscle of *Ryr1*^TM/indel^ mice (Brennan et al., 2019). Indeed, following normalization to muscle mass, twitch contraction remained reduced in *Ryr1*^TM/indel^ mice compared to that in WT mice. This result indicates that other intrinsic parameters of the muscle than the reduced muscle mass were involved in the decreased force production, such as the decreased Ca^2+^ release. However, the normalized maximal tetanic force in *Ryr1*^TM/indel^ mice was similar to that in WT mice, which may emphasize that muscle weakness (i.e. reduction in maximal force) in *Ryr1*^TM/indel^ mice was mainly related to reduced skeletal muscle mass. Our result differs from those of Brennan et al. (2019), who reported lower maximal specific force, indicating that muscle weakness is independent of reduced muscle mass. However, although experiments were performed at the same age, we studied TA muscle *in situ*, whereas Brennan et al. (2019) studied the extensor digitorum longus muscle *ex vivo*. The discrepancy between these results may be related to the type of muscle and/or to the technique of force measurements. Interestingly, submaximal tetanic contraction (i.e. first contraction at 40 Hz) was also not affected by the mutation. Therefore, apart from the difference in muscle/technique used, we could hypothesize extracellular Ca^2+^ entry as a compensatory mechanism to contribute to tetanic contractile force production (Cho et al., 2017). For both twitch and tetanic contractions, the time to maximum was similar between groups, which suggests that Ca^2+^ transients may be unaltered by the mutation or the treatment. Therefore, the reduced rate of contraction seems to be related to the lower force produced. Following tetanic contraction, the half relaxation time was significantly increased in *Ryr1*^TM/indel^ mice, illustrating decreased capacities and/or rate in Ca^2+^ repumping into the SR in *Ryr1*^TM/indel^ mice during tetanic contractions. During fatigue, the drop in force and the rate of relaxation during repetitive contractions were similar in WT and *Ryr1*^TM/indel^ groups, indicating that muscle fatigue was unaltered in *Ryr1*^TM/indel^ mice. In addition, force production throughout the fatigue protocol was unaffected by the mutation despite increased half relaxation time over time, which may potentially be caused by aberrant Ca^2+^ uptake to the SR in *Ryr1*^TM/indel^ mice.

The main histological feature observed in CNMs is the mispositioning of organelles. Accumulation of mitochondria within muscle fibers either in the center or at the periphery is typically observed in mouse models of CNMs (Durieux et al., 2010; Munoz et al., 2020; Silva-Rojas et al., 2022; Buj-Bello et al., 2002). However, unlike these CNM mice, *Ryr1*^TM/indel^ mice did not display this defect. Nuclei are also commonly found either internalized or centrally located within muscle fibers in CNM mice (Brennan et al., 2019; Durieux et al., 2010; Munoz et al., 2020; Buj-Bello et al., 2002). *Ryr1*^TM/indel^ mice showed nuclei positioning anomalies in ∼5% of the fibers, whereas Brennan et al. (2019) reported <1% of affected myofibers. The type of analysis (mislocalized in the present study versus centralized in the previous study) likely explains the difference in quantification considering that same muscle was analyzed at the same age in both studies. Nevertheless, our result is consistent with what was reported in other models of CNMs (Gineste et al., 2023; Swamynathan et al., 2024). Abnormal localization of nuclei and mitochondria were observed in patients with CNM with *RYR1* mutations, indicating that the *Ryr1*^TM/indel^ model partly reproduces the histological features observed in human (Jungbluth et al., 2007; Wilmshurst et al., 2010; Neto et al., 2017).

The pathological role of DNM2 in *MTM1*- *DNM2*- and *BIN1*-related forms of CNM was demonstrated in mouse models. Indeed, DNM2 levels were largely increased in the muscles of mouse models for these three forms of CNM, as well as in patients with *MTM1*-related CNM (Gineste et al., 2023; Cowling et al., 2014; Koch et al., 2020). This increase in DNM2 is pathological in CNM considering that its decrease improves the phenotype of models of different forms of CNM (Tasfaout et al., 2017; Munoz et al., 2020; Silva-Rojas et al., 2022). Here, we detected a non-significant trend towards increased DNM2 protein levels in *Ryr1*^TM/indel^ mice, suggesting that increased DNM2 levels are not a common feature of different CNM forms and that *RYR1*-related CNM might not be driven by increased DNM2 levels. However, DNM2 expression in patients remains to be confirmed, pending access to such samples, to elucidate the potential role of DNM2 in the pathogenesis of *RYR1*-related CNM.

### Effects of tamoxifen treatment on *Ryr1*^TM/indel^ mice

Our results showed no improvement of phenotype at functional, histological and molecular levels in *Ryr1*^TM/indel^ mice following tamoxifen treatment, in contrast to previous data on other preclinical models of CNMs linked to mutations in genes other than *RYR1*. This absence of phenotype amelioration was not related to a decrease in tamoxifen intake, given that tamoxifen intake was similar in *Ryr1*^TM/indel^ and WT mice. Moreover, we reported similar amounts of tamoxifen metabolites in muscles from treated *Ryr1*^TM/indel^ and WT mice, which indicates that the activities of the main cytochrome P450 (CYP) enzymes involved in tamoxifen metabolism are unaltered. Nevertheless, we observed that the amount of 4-hydroxytamoxifen tended to be doubled (*P*=0.06) in *Ryr1*^TM/indel^ muscle compared to WT muscle. Our data indicate that the activity of other CYP isoforms may be increased by the mutation, leading to accumulation of 4-hydroxytamoxifen in *Ryr1*^TM/indel^ mice. However, genetic variants directly affect the activity of CYP enzymes (Cronin-Fenton et al., 2014; Lyon et al., 2012; Jin et al., 2005; Kanji et al., 2023). Therefore, it cannot be excluded that the enzymes involved in tamoxifen metabolism are altered in *Ryr1*^TM/indel^ mice.

The *Ryr1*^TM/indel^ mouse model did not show any improvement in the *in vivo* phenotype with tamoxifen intake, as illustrated by the similar spontaneous locomotor and rearing activities at 6 and 8 weeks of age in untreated and tamoxifen-treated *Ryr1*^TM/indel^ groups. Following tamoxifen administration, twitch and maximal forces were unchanged in *Ryr1*^TM/indel^ mice. Our present results are in agreement with our previous data in the mouse model of *DNM2*-related CNM but contrast with those reported in the *MTM1*- and *BIN1*-related CNM forms (Gineste et al., 2023; Gayi et al., 2018; Maani et al., 2018). As previously suggested, the effect of tamoxifen on maximal force may be associated with a specific pathomechanism. During repetitive submaximal contraction, despite the lower force produced, the decline in force and the rate of relaxation during repetitive contractions were unchanged, as previously observed in other genetic models of CNMs treated with tamoxifen (Gineste et al., 2023). It is well known that tetanic intracellular Ca^2+^ concentration and Ca^2+^ sensitivity decrease particularly during fatigue-induced submaximal contractions (Allen et al., 2008; Place et al., 2010). However, both parameters were unlikely differently altered between genotypes considering that fatigue properties were similar between WT and *Ryr1*^TM/indel^ groups. Additionally, the lack of difference in fatigue development in tamoxifen-treated *Ryr1*^TM/indel^ mice compared to untreated *Ryr1*^TM/indel^ mice can be explained by a reduced energy cost of the contraction associated with the lower Ca^2+^ release and force production (Cheng et al., 2019). We did not report any difference in the contractile parameters between tamoxifen-treated and untreated *Ryr1*^TM/indel^ mice, except for a decreased half relaxation time following maximal tetanic contraction and repetitive submaximal contractions. Our data indicate an effect of tamoxifen on Ca^2+^ reuptake following tamoxifen administration, although the underlying mechanism requires further investigation. Although this improvement could suggest better Ca^2+^ handling following tamoxifen administration, it had no positive impact on muscle force production in *Ryr1*^TM/indel^ mice.

The levels of DNM2 and BIN1 proteins were assessed to investigate molecular changes following tamoxifen treatment. Interestingly, the abnormally high DNM2 levels were previously found decreased following tamoxifen administration in mouse models of *MTM1*-, *DNM2*- and *BIN1*-related CNM and most probably account for the phenotypic improvement (Gineste et al., 2023; Gayi et al., 2018; Maani et al., 2018). Here, we observed that tamoxifen did not change the level of DNM2 in muscles of *Ryr1*^TM/indel^ mice or WT mice. These data suggest that the lack of phenotypic rescue following tamoxifen treatment may be related to the inability to decrease DNM2 levels. Our results also imply that DNM2 levels may be decreased by tamoxifen when they are abnormally elevated, given that we previously reported that tamoxifen did not alter DNM2 levels in WT mice, regardless of the dose (Gineste et al., 2023). Interestingly, Maani et al. (2025) demonstrated that microRNA-133a (miR-133a) was upregulated in response to treatments improving DNM2 levels, including tamoxifen therapy, which may explain how tamoxifen decreases *Dnm2* levels in several models. Although miR-133a downregulation has not been demonstrated or thoroughly examined in *Ryr1*^TM/indel^ mice, it could also potentially account for the lack of responsiveness to tamoxifen in this model. On the other hand, our data correlate nicely with the findings of Onofre-Oliveira et al. (2025) who recently showed that lowering *Dnm2* with ASO did not improve the phenotype in *Ryr1*^TM/indel^ mice.

Finally, a lack of effect of tamoxifen on the phenotype of *Ryr1*^TM/indel^ mice was observed in both males and females. The effectiveness of tamoxifen previously reported in models of CNMs varied and was likely not dependent on sex or ERα levels alone (Gayi et al., 2018; Maani et al., 2018). Modest improvements were seen in male *MTM1*-related CNM mice and in male and female *DNM2*-related CNM mice (with 5-fold and 1.5-fold ERα increase, respectively), while complete rescue occurred in male and female *BIN1*-related CNM mice (with a 1.5-fold ERα increase). Overall, our data indicate that the absence of beneficial effect in *Ryr1*^TM/indel^ mice was not sex related.

### Potential adverse effects of tamoxifen administration

Importantly, our results demonstrated impairment of muscle contractility following tamoxifen treatment. Tamoxifen-treated WT mice displayed muscle weakness compared to the untreated WT mice (−23%) related to muscle mass reduction. Interestingly, the detrimental effect of tamoxifen on muscle mass was more severe in males than in females (−30% versus −15%, respectively). The muscle mass reduction in tamoxifen-treated WT mice, which was already reported in our previous study (Gineste et al., 2023), may result from an imbalance in muscle protein turnover, potentially triggered by a decrease in estrogen or ERα levels, or tamoxifen acting as an antagonist (Collins et al., 2019; Ikeda et al., 2019). We did not observe such effect on muscle mass in *Ryr1*^TM/indel^ mice, likely because the muscle mass is already largely reduced.

In *Ryr1*^TM/indel^ mice, force production was lower during submaximal contractions following tamoxifen administration compared to that in untreated *Ryr1*^TM/indel^ mice. Our data highlight a negative effect of tamoxifen on muscle function during submaximal contractions, with a consequential unfavorable impact on whole-body performance. Indeed, we observed a decrease in rearing activity over time in tamoxifen-treated *Ryr1*^TM/indel^ mice, which likely represents a progression of whole-body muscle weakness with tamoxifen administration. Our findings are in sharp contrast with previously reported results showing large benefits of tamoxifen on force generation during submaximal contractions in preclinical mouse models of CNM, with mutations in *Mtm1*, *Bin1* and *Dnm2* genes, as well as in dystrophic mice (Gineste et al., 2023; Gayi et al., 2018; Maani et al., 2018; Dorchies et al., 2013). The reduction in submaximal force was independent of decreased muscle mass. Therefore, the decreased force-generating ability during repetitive contractions in *Ryr1*^TM/indel^ mice after tamoxifen administration is likely to be due to other intrinsic properties of the muscle. The main cause of muscle force decrease in *Ryr1*^TM/indel^ mice is the reduction of Ca^2+^ release during muscle contraction, which may be worsened by tamoxifen administration, particularly considering that the amount of Ca^2+^ released directly influences the amount of force generated (Gineste et al., 2023).

### Conclusions

In conclusion, we found that the muscle weakness of *Ryr1*^TM/indel^ mice results mainly from reduction in muscle mass and myofiber size, and demonstrated that tamoxifen did not antagonize the CNM-like disease in *Ryr1*^TM/indel^ mice. Moreover, we observed worsening of muscle contractility upon tamoxifen supplementation. Overall, our data imply that tamoxifen may not serve as a therapeutic approach for patients with CNM with *RYR1* mutations.

## MATERIALS AND METHODS

### Animals, treatment and study approval

Male and female compound heterozygote mice carrying a T4709M missense mutation (TM allele) and a 16 bp frameshift deletion (indel allele) in *Ryr1* gene (*Ryr1*^TM/indel^ mice) were generated and identified through PCR genotyping from mouse ear DNA. WT littermates were used as controls. In order to optimize the distribution of mice in each group, experiments were not conducted unaware of the genotypes and drug. For each litter and after genotypes were confirmed, WT and *Ryr1*^TM/indel^ mice were allocated to the untreated group or to the tamoxifen treatment group. The mouse line was maintained on a pure C57BL/6J background.

*Ryr1*^TM/indel^ and WT mice were exposed to either a tamoxifen-enriched diet (tamoxifen treatment group) or a standard rodent diet without tamoxifen (untreated group) from 3 weeks (weaning at 21 days) until 8 weeks (i.e. 56 days) of age. Tamoxifen citrate (T9262, Sigma-Aldrich) was added to the standard rodent diet to reach 65 mg/kg of food. Food consumption per cage and body weight was measured every week in order to confirm tamoxifen-enriched food intake. The amount of food eaten per day per mouse was divided by the weight of each individual mouse to calculate tamoxifen intake.

All experiments were conducted in agreement with the French guidelines for animal care and in conformity with the European legislation for the use of animals for scientific purposes and approved by the Institutional Ethic Committee (permit number 38760-2022100215534821). Mice were housed in an environment-controlled facility (12 h-12 h light-dark cycle, 22°C) and received water and food *ad libitum*.

### Longitudinal assessment of disease progression

Body mass was measured weekly from the start of treatment (i.e. 3 weeks) until the end of the treatment (i.e. 8 weeks). Global activity level was assessed at 6 and 8 weeks, i.e. 3 and 5 weeks after the start of treatment, with the open-field test. Mice were placed individually in an automated arena of 44 cm (width)×44 cm (length)×17 cm (height) made of polyvinyl chloride (PVC) with translucent walls, a black floor and covered with translucent PVC and fitted with two frames of 32 infrared beams (Panlab). The open-field arenas were located in a homogeneously illuminated room (150±15 Lux). Horizontal locomotor activity and vertical activity of each mouse were monitored within the whole field for 30 min starting when the mouse was introduced in the arena. Data were acquired with ActiTrack software (Panlab).

### *In situ* force output measurements

At the end of the treatment period, 8-week-old mice were anesthetized through a triple shot cocktail by intraperitoneal injection of domitor/fentanyl mix (2/0.28 mg/kg), diazepam (8 mg/kg) and fentanyl (0.28 mg/kg). The right TA muscle was exposed, and the distal tendon was cut and secured to a hook, which was connected to the level arm of the 809B mouse apparatus (Aurora Scientific). The knee joint was clamped, and the ankle joint was restrained to stabilize the limb. A 2-3 mm segment of the sciatic nerve was accessed with an incision made on the lateral side of the thigh and freed from surrounding tissue. Needle electrodes were inserted and connected directly to the nerve. TA muscle contractions were induced by delivering electric pulses (0.2 ms duration) to the sciatic nerve. The optimal resting length of the muscle, defined as the length at which the muscle generates the maximum isometric twitch force, was determined by gradually extending the muscle until the maximum peak twitch force was reached. The maximal stimulation intensity was determined by progressively increasing the stimulus intensity until no further increase in peak twitch force was observed. TA twitch and tetanic muscle forces were measured at 1 Hz and 150 Hz, respectively (0.5 s). TA force was then measured during a fatigue protocol consisting of 80 contractions at 40 Hz (1 s on, 3 s off). Measurements were conducted using the 150A-305 Integrated Muscle Test Controller (Aurora Scientific). The protocols were controlled and data were recorded on a personal computer using 610A DMC software (Aurora Scientific).

### Tissue collection

At the end of the force experiments, hindlimb muscles (Gas, Sol and TA) from 8-week-old mice were excised, weighed and frozen in liquid nitrogen-cooled isopentane before being stored at −80°C.

### Histology

Transverse cryosections (8 μm) of TA muscles sampled from 8-week-old mice were collected on glass slides and stained with Hematoxylin-Eosin (HE) or SDH. For HE staining, frozen sections were dried for 10 min at room temperature. Sections were then fixed in refrigerated acetone for 10 s. Sections were dried in an oven at 37°C for 2 h before being hydrated in tap water for 30 s. Slides were incubated in Harris Hematoxylin for 3 min and then washed in tap water for 3 min. Sections were decolorized in acid alcohol for 2 s and then washed and blue colored in tap water for 3 min. Slides were incubated in 0.1% aqueous Eosin Y for 30 s and rinsed in tap water for 30 s. Sections were dehydrated in graded alcohols, 2×2 min in 95% alcohol followed by 2×2 min in 100% alcohol. Sections were cleared in Histosol 2×2 min. Slides were mounted after excess Histosol was evaporated by using two drops of mounting medium (Eukitt) and by placing a coverglass (25×60 mm) on the slide. Slides were dried overnight at room temperature. For SDH staining, slides were incubated in freshly made staining solution (5 ml of 0.2 M potassium dihydrogen phosphate, 19 ml of 0.2 M di-sodium hydrogen phosphate, 25 ml of sodium succinate solution and 1 ml Nitro Blue tetrazolium solution, pH adjusted to 7.35 with 1 M sodium hydroxide) for 1 h at 37°C. Slides were washed in PBS 3×5 min before being postfixed in 10% buffered formalin solution for 10 min. Slides were rinsed in 15% ethanol 2×5 min and then mounted with aqueous mounting medium.

### Protein extraction and western blotting

TA muscles from 8-week-old mice were homogenized in RIPA buffer containing 1 mM PMSF, 1 mM DTT, 1 mM sodium orthovanadate, 5 mM sodium fluoride and a complete EDTA-free protease inhibitor cocktail (Roche Diagnostics) using a Precellys 24 tissue homogenizer (Bertin Technologies). The DCTM Protein Assay Kit (Bio-Rad) was used to measure protein concentrations. Samples were denatured by heating for 5 min at 95°C with 5× Lane Reducing Buffer (Thermo Fisher Scientific) and loaded onto a 6% SDS-PAGE gel. Proteins were transferred to nitrocellulose membranes using a Transblot TurboTM RTA Transfer Kit (Bio-Rad). Protein loading was verified by Ponceau Red staining. Membranes were blocked for 1 h in TBS with 5% non-fat dry milk and 0.1% Tween 20, followed by overnight incubation at 4°C with primary antibodies. Membranes were then incubated with secondary antibodies conjugated to horseradish peroxidase for 1 h at room temperature. The primary antibody used was anti-pan-DNM2 (rabbit, 1:1000, 2865; homemade) or anti-pan-BIN1 (rabbit, 1:1000, 3623; homemade). A goat anti-rabbit secondary antibody was used (1:10,000 for pan-DNM2 and 1:5000 for pan-BIN1, 111-036-045, Jackson ImmunoResearch).

### Quantification of tamoxifen and its metabolites

The concentrations of tamoxifen and the metabolites of tamoxifen (OH-tamoxifen, N-desmethyl-tamoxifen and endoxifen) in Gas muscles collected from 8-week-old mice were measured using high-performance liquid chromatography coupled to tandem mass spectrometry as previously described (Gineste et al., 2023).

### Data processing

#### Open field

Animal trajectories (distance covered) and rearings in the whole arena during the 30-min recording period were analyzed using ActiTrack software (Panlab).

#### Contractile performance

Data were analyzed using 611A DMA and 612A DMA-HT software (Aurora Scientific). The maximal force (i.e. the peak force produced), time to maximum (i.e. the time to reach the peak force from stimulation) and half relaxation time (i.e. time to reach 50% of relaxation from the end of stimulation), as well as the rates of contraction and relaxation [i.e. the slope of the ascending or descending force traces between 20-80% of the contraction or relaxation (average rate) or 0-100% (maximum rate)], of both twitch and tetanic contractions were measured. For clarity, the maximum, half relaxation time and rate of relaxation were plotted every five contractions for the fatigue protocol. Total force production was calculated as the sum of the individual peak forces measured during the fatigue protocol. The drop in force (i.e. force decline) was calculated as the difference (in %) in force between the last and the first contraction of the fatiguing protocol. The time to fatigue corresponds to the time to drop 30% of the force recorded at the first contraction of the fatigue protocol. Specific force (in mN/mg) was determined by normalizing the force to the TA muscle mass.

#### Imaging

Light microscopic images of HE and SDH sections were acquired using a Nanozoomer 2HT slide scanner (Hamamatsu Photonics). A homemade FIJI plugin was used to perform fiber size analysis. The cell counter plugin in FIJI analysis software was used to perform analyses of abnormally positioned nuclei. Myofiber minimum feret (MinFeret) diameter (µm) and the percentage of fibers with mislocalized nuclei were determined in the whole-muscle sections.

#### Western blotting

Nitrocellulose membranes were scanned in an Amersham Imager 600 (GE Healthcare Life Sciences). Band signal intensities were determined using FIJI software. The quantified levels of proteins were normalized to Ponceau Red and expressed as the fold difference from those of the untreated WT group.

### Statistical analysis

Data are presented as individual data points and mean±s.e.m. or as mean±s.e.m. Statistical analyses were performed with GraphPad Prism software version 10.2.2. Normality was checked using a Shapiro–Wilk test. Parametric tests were used for normally distributed data. Non-parametric tests were used otherwise. Mixed-effects analysis was used to compare body weight and tamoxifen intake over time. When a significant interaction was found, Tukey's post hoc test was performed. Student’s unpaired two-tailed *t*-test was used to compare the levels of tamoxifen metabolites in WT and *Ryr1*^TM/indel^ mice. One-way ANOVA or Kruskal–Wallis test was used for all other comparisons, followed by Tukey's post hoc test or Dunn's post hoc test, respectively, when a significant difference was observed. Statistical significance was accepted when *P<*0.05.

## Supplementary Material

10.1242/dmm.052462_sup1 Supplementary information
