# Supplementary material for: Tamoxifen treatment fails to improve muscle dysfunction in a model of recessive RYR1-linked centronuclear myopathy
Source: Dis Model Mech. 2025 Dec 29;18(12):dmm052462. doi: 10.1242/dmm.052462 (PMC12805646; doi:10.1242/dmm.052462)
Supplement: Supplementary information [file dmm-18-052462-s1.pdf]

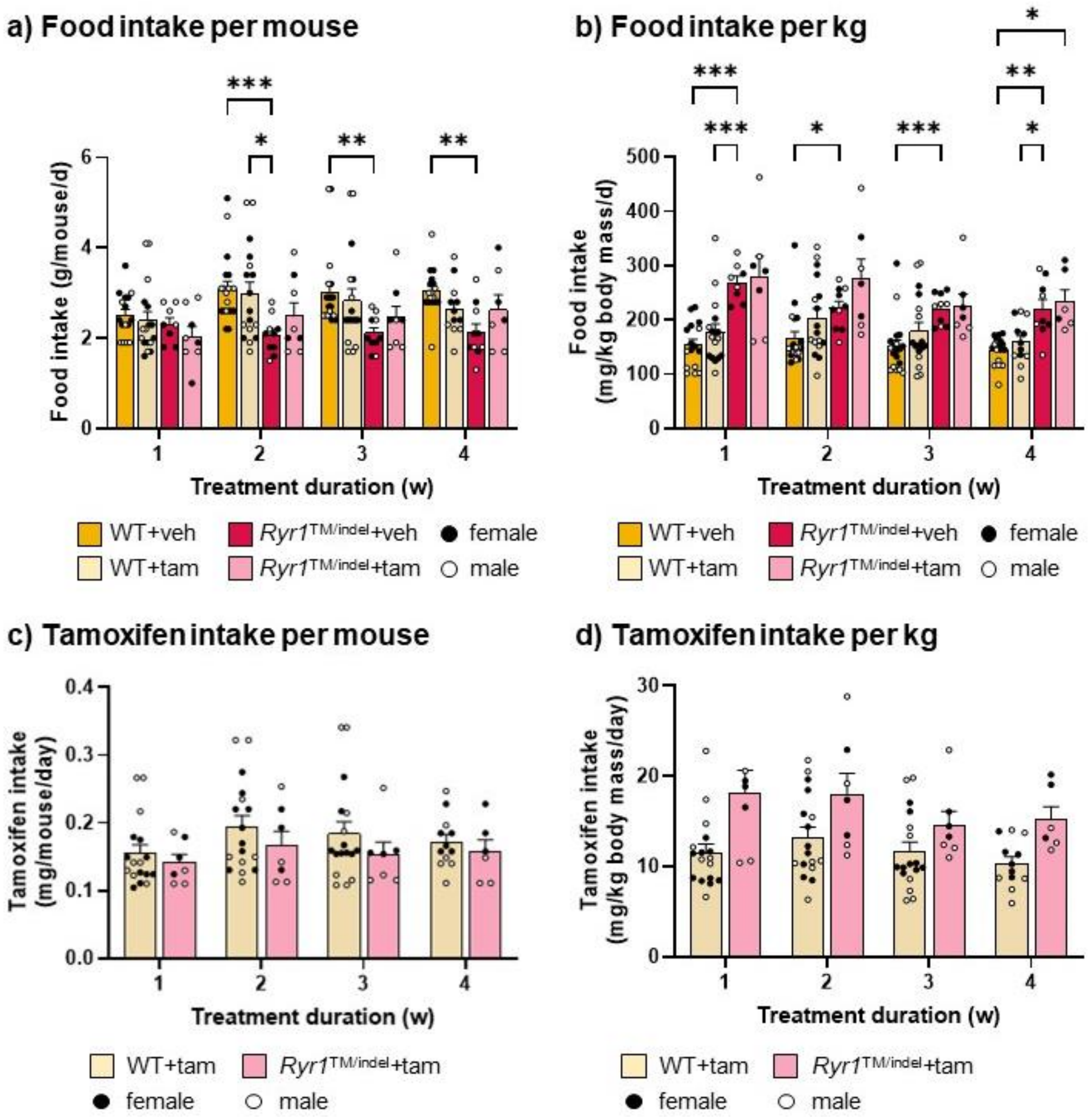

Fig. S1. Food (panels a and b) and tamoxifen (panels c and d) intake of *Ryr1*$^{TM/indel}$ and WT mice throughout the 4 first week of intervention. Food consumption and tamoxifen intake was calculated by dividing the amount of food eaten per day per mouse by the weight of each individual mouse. *p*-values obtained with mixed-effects analysis with Tukey's post hoc test.

Disease Models & Mechanisms • Supplementary information

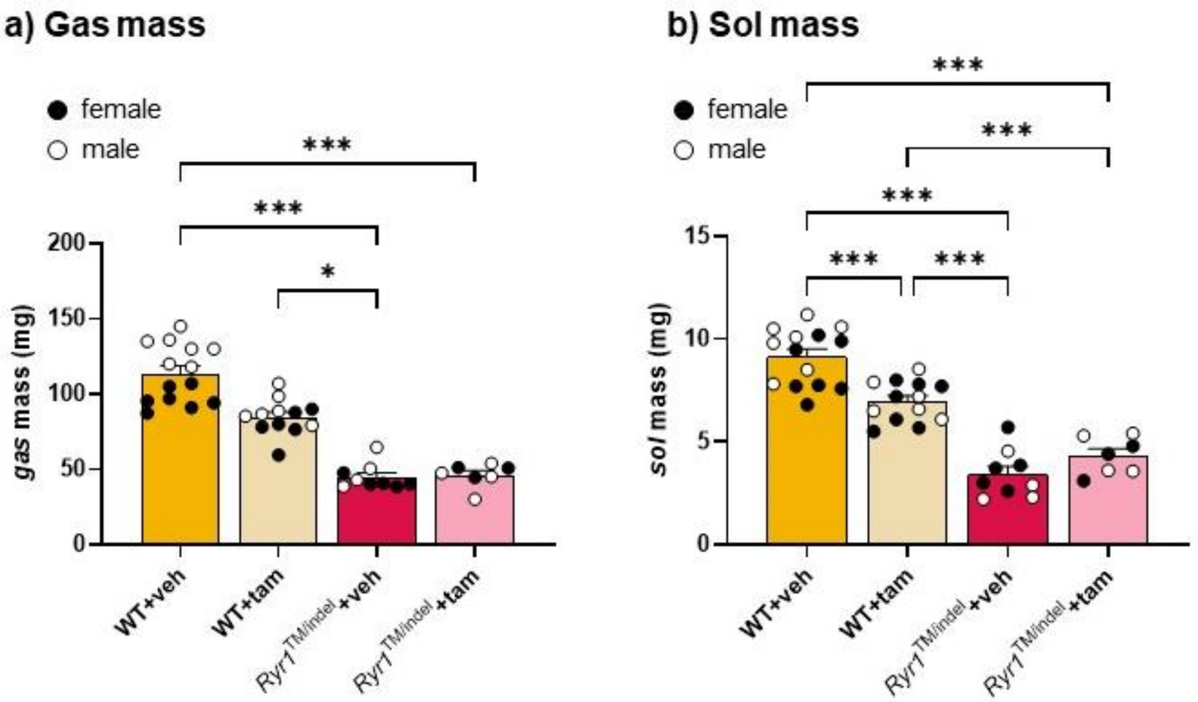

Fig. S2. Mass of gas (a) and Sol (b) from non-treated and tamoxifen-treated *Ryr1*$^{TM/indel}$ and WT mice following 5-week exposure to a standard diet or a tamoxifen-enriched diet. Data presented as individual values and mean ± SEM. *p*-values obtained with one-way ANOVA Kruskal-Wallis with Dunn's post hoc test (panel a-b). Significant difference *, ** and *** $p <$ 0.05, 0.01 and 0.001, respectively.

Disease Models & Mechanisms • Supplementary information

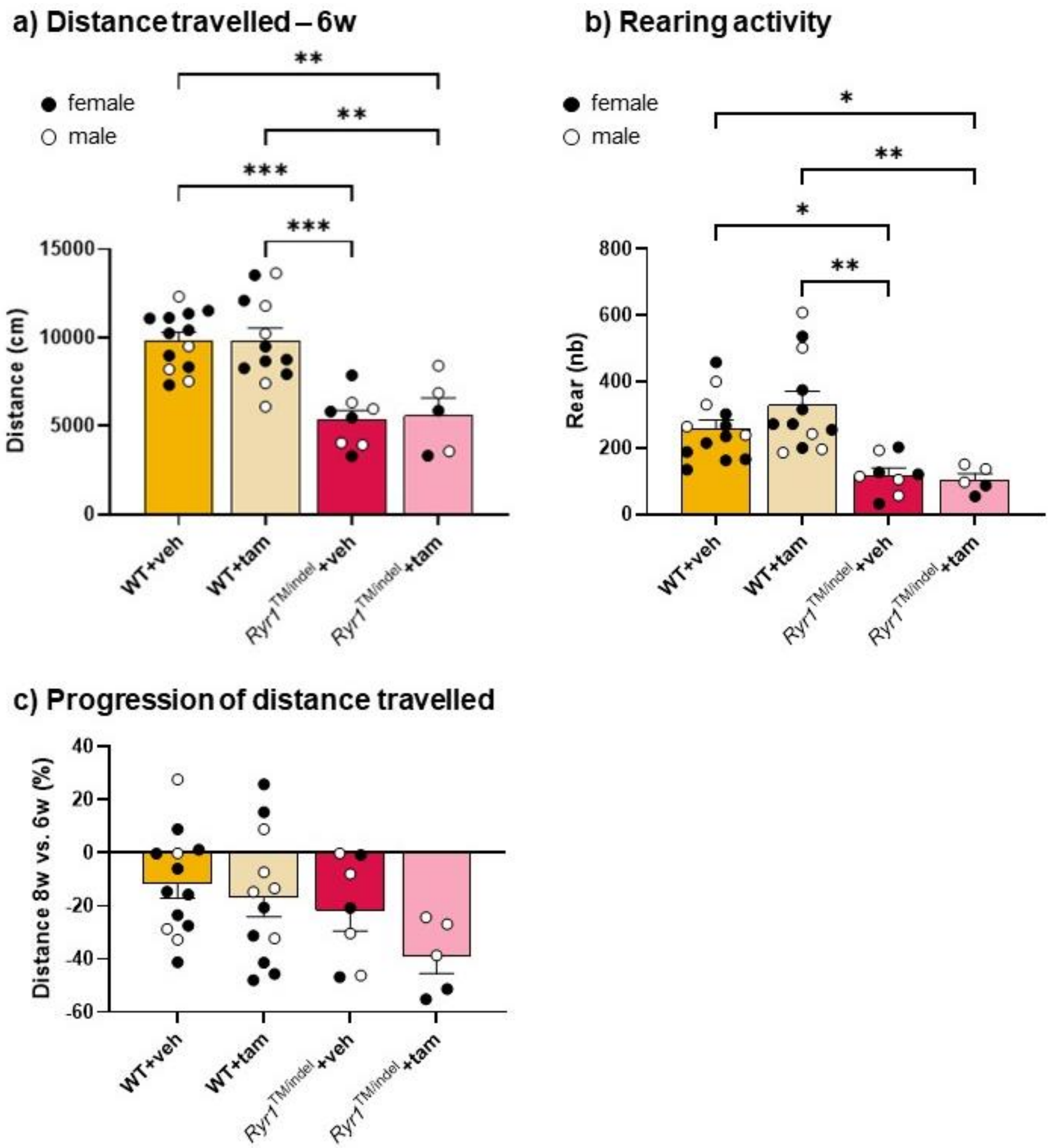

Fig. S3. Distance covered (a) and number of rears (b) during 30-min period in *Ryr1*$^{TM/indel}$ and WT groups at 6 weeks of age. (d) Changes in spontaneous locomotor activity between 6 weeks of age and 8 weeks of age, i.e. 3 and 5 weeks after treatment intervention start, in *Ryr1*$^{TM/indel}$ and WT groups. Data presented as individual values and mean ± SEM. *p*-values obtained with one-way ANOVA with Tukey's post hoc test (panel a and c) or Kruskal-Wallis with Dunn's post hoc test (panel b). Significant difference *, ** and *** $p < 0.05$, 0.01 and 0.001, respectively.

Disease Models & Mechanisms • Supplementary information

Table S1. Levels of the three major metabolites of tamoxifen (4-hydroxytamoxifen, N- desmethyltamoxifen, and endoxifen) in gastrocnemius muscles of *Ryr1*$^{TM/indel}$ and WT mice exposed to tamoxifen-enriched diet for 5 weeks. *p*-values obtained with two-tailed unpaired *t*-test.

Metabolite	Gas		*P*-value
	WT	*Ryr1*$^{TM/indel}$	
**N-desmethyltamoxifen (ng/g)**	1.28±0.27	1.71±0.30	0.31
**4-hydroxytamoxifen (ng/g)**	9.59±2.35	22.77±6.52	0.06
**E-endoxifen (ng/g)**	4.85±0.68	4.75±0.83	0.93
**Z-endoxifen (ng/g)**	4.40±1.03	5.73±1.10	0.40
**Total (ng/g)**	20.12±4.33	34.96±8.75	0.49

Disease Models & Mechanisms • Supplementary information
